# Whole‐body MRI for cancer surveillance in ataxia–telangiectasia: A qualitative study of the perspectives of people affected by A‐T and their families

**DOI:** 10.1111/hex.13756

**Published:** 2023-03-16

**Authors:** Renata Neves, Blanca de Dios Perez, Tierney Tindall, Natasha Schneider Fernandez, Rafal Panek, Sophie Wilne, Mohnish Suri, William Whitehouse, Sumit Jagani, Madhumita Dandapani, Robert A. Dineen, Cris Glazebrook

**Affiliations:** ^1^ Radiological Sciences, Mental Health and Division of Clinical Neuroscience University of Nottingham Nottingham UK; ^2^ Department of Radiology Nottingham University Hospitals NHS Trust Nottingham UK; ^3^ Division of Rehabilitation, Ageing and Wellbeing, Centre for Rehabilitation and Ageing Research, School of Medicine University of Nottingham Nottingham UK; ^4^ Mental Health and Division of Clinical Neuroscience, School of Medicine University of Nottingham Nottingham UK; ^5^ Independent Patient and Parent Representative Nottingham UK; ^6^ Department of Medical Physics and Clinical Engineering Nottingham University Hospitals NHS Trust Nottingham UK; ^7^ Department of Paediatric Oncology Nottingham University Hospitals NHS Trust Nottingham UK; ^8^ Nottingham Clinical Genetics Service Nottingham University Hospitals NHS Trust Nottingham UK; ^9^ Division of Child Health, Obstetrics and Gynaecology, School of Medicine University of Nottingham Nottingham UK; ^10^ Department of Radiology, Nottingham Children's Hospital Nottingham University Hospitals NHS Trust Nottingham UK; ^11^ NIHR Nottingham Biomedical Research Centre Nottingham UK; ^12^ Children's Brain Tumour Research Centre, Medical School University of Nottingham Nottingham UK; ^13^ Division of Clinical Neuroscience, Sir Peter Mansfield Imaging Centre University of Nottingham Nottingham UK; ^14^ Institute of Mental Health University of Nottingham Nottingham UK

**Keywords:** cancer predisposition, life‐limiting disease, psychosocial impact, Public and Patient Involvement and Engagement, qualitative research

## Abstract

**Background/Objectives:**

Ataxia–telangiectasia (A‐T) is a complex inherited disease associated with an increased risk of malignancy. Surveillance guidelines have demonstrated significant health benefits in other cancer predisposition syndromes. However, evidence‐based guidelines for cancer screening are not currently used in the United Kingdom for people affected by A‐T. This study aims to understand how people with A‐T and their parents feel about cancer surveillance using whole‐body magnetic resonance imaging (MRI) to inform the future development of cancer surveillance guidelines.

**Design/Methods:**

We conducted semistructured interviews with people affected by A‐T. Data were analysed inductively using thematic analysis.

**Results:**

Nine parents of children with A‐T and four adults with A‐T were interviewed. Five main themes emerged from the data, including (1) cancer screening was considered invaluable with the perceived value of early detection highlighted; (2) the cancer fear can increase anxiety; (3) the perceived limitations around current practice, with the responsibility for monitoring falling too strongly on parents and patients; (4) the need for effective preparation for cancer screening, including clear communication and (5) the challenges associated with MRI screening, where specific recommendations were made for improving the child's experience.

**Conclusion:**

This study suggests that stakeholders are positive about the perceived advantages of a cancer screening programme. Ongoing support and preparation techniques should be adopted to maximise adherence and minimise adverse psychosocial outcomes.

**Patient or Public Contribution:**

People with A‐T and parents of people with A‐T were actively involved in this study by giving their consent to be interviewed. An independent parent representative contributed to the study, supporting the research team in interpreting and commenting on the appropriateness of the language used in this report.

## 1 INTRODUCTION

Ataxia–telangiectasia (A‐T) is a rare inherited disease that results in cerebellar neurodegeneration, elevated cancer risk, radiation sensitivity, immunodeficiency and respiratory disease.^1^, ^2^, ^3^ The estimated prevalence of A‐T in the United Kingdom is approximately 1 in 400,000,^4^ with 170–200 people affected.^4^, ^5^ A‐T is a progressive condition that manifests in early childhood.^2^ The life expectancy of people with A‐T is estimated to be 25 years, with cancer being the most frequent reason for mortality.^2^, ^5^, ^6^, ^7^ Cancer in A‐T has been reported as early as 2 years old with a median age of diagnosis of 12.5 years.^8^, ^9^

Cancer surveillance guidelines for individuals affected by some cancer predisposition syndromes (CPS) with elevated cancer risk, such as Li–Fraumeni syndrome (LFS), have been developed and can help to reduce delays to diagnosis and initiation of treatment.^10^, ^11^ Despite the high cancer risk that people with A‐T experience (22%–24% cumulative incidence up to age 20 years), current guidelines for the management of children and young adults with A‐T do not include cancer surveillance.^2^, ^4^, ^8^, ^9^, ^12^ An international consensus statement regarding screening in CPS noted that ‘Evidence‐based standards for cancer screening do not exist for patients with A‐T, particularly in childhood’.^10^

Developments in magnetic resonance imaging (MRI) technology allow whole‐body (WB)‐MRI scans to be performed with relatively short acquisition times.^13^, ^14^, ^15^, ^16^, ^17^, ^18^, ^19^, ^20^, ^21^ WB‐MRI protocols optimised for cancer detection are used clinically for diagnosing and monitoring sarcomas, metastases and haematological tumours like myeloma.^22^, ^23^, ^24^, ^25^ Previous trials have explored the use of WB‐MRI along with other diagnostic tests to develop guidelines for cancer surveillance in CPS.^17^, ^19^, ^26^, ^27^, ^28^, ^29^ The excellent soft‐tissue contrast resolution and ability to demonstrate malignancy contribute to the increased interest in including this technique in cancer surveillance protocols.^17^, ^18^, ^19^, ^20^, ^21^, ^30^ However, it is important to consider the challenges associated with this technique, such as the relatively long imaging times and image artefacts caused by motion, which may require the use of sedation or general anaesthesia in young children.^17^, ^31^ The high sensitivity of MRI might detect incidental findings (false positives), which could lead clinicians to request unnecessary examinations.^20^ Nevertheless, trials of cancer surveillance in families with LFS that include WB‐MRI have demonstrated the feasibility of the approach, with significantly improved survival (thus better outcomes), and WB‐MRI is now incorporated into clinical guidelines.^27^, ^28^, ^29^, ^32^, ^33^, ^34^, ^35^ The lack of ionising radiation in MRI makes this an attractive approach for imaging‐based cancer surveillance in people with A‐T, but this approach has not yet been systematically evaluated or incorporated into guidelines for people with A‐T. In practice, WB‐MRI would be combined with blood tests (e.g., to pick up leukaemia) in a surveillance programme.

People with A‐T and their families already have the worry of living with a chronic life‐limiting childhood‐onset disease with a poor overall prognosis and progressive physical disability.^1^, ^2^, ^3^ A cancer surveillance programme could add to this burden with a detrimental effect on both the child with A‐T and their family members. It is recognised that surveillance tests can increase anxiety due to the possibility of finding cancer or other pathologies.^20^ Data from screening trials related to other CPS have demonstrated mixed psychosocial effects: although anxiety, depression and cancer worry were reported, overall the levels of satisfaction and acceptability were higher.^36^, ^37^, ^38^ These trials have shown that having clinical support to provide psychosocial care, such as a long‐term framework of clinical consultation, peer support scheme either by telephone or email and support network with educational support groups, is as important as the cancer screening itself.^27^, ^28^, ^29^, ^32^ Quality‐of‐life is affected by A‐T,^39^ and thus it is important to understand whether cancer screening would increase the levels of anxiety and have additional negative impacts on the quality‐of‐life of people with A‐T and their families.

To date, no study has explored the perceptions of people with A‐T and their families regarding cancer screening and what support would be beneficial to improve the acceptability of this programme. Therefore, this study seeks to understand the views of people with A‐T and their parents regarding the idea of an MRI‐based cancer surveillance programme before a planned feasibility trial of WB‐MRI scans for cancer screening in children and young people with A‐T.

## 2 METHODS

### 2.1 Participants

The target sample size was 15 participants to capture a diverse range of views and ensure data saturation.^40^ We invited people with A‐T and parents (mothers and fathers) of people with A‐T to participate through adverts published on the social media channels of the two A‐T UK charities. These charities also contacted their members by email with a brief description of the study. Information sheets, which included a video explaining the study (provided in Supporting Information: Material S1), were sent to everyone who showed interest in the study by emailing the research team. Participants confirming that they had read the participant information sheet were sent a link to complete the electronic consent form and provide demographic information. Participants were recruited between July and November 2021.

Participants with A‐T were included if they had a formal diagnosis of A‐T and were over the age of 12. The inclusion criterion for parents was to have, or have had, a child with A‐T. This study was approved by the University of Nottingham Research Ethics Committee (Ref: 2787), and participants gave electronic consent before the interview.

### 2.2 Data collection

The semi‐structured interviews were conducted by two research team members (R. N. and B. D. P.). The interviews were directed by a topic guide, which was tailored to parents of people with A‐T and people affected by A‐T (see example questions in Supporting Information: Materials S2 and S3). The interview questions explored the potential impact that a cancer screening programme could have on children and young people affected by A‐T and their families, considering the current practice and the cancer risk in A‐T. We also discussed the use of MRI as a screening technique. The interviews were conducted on Microsoft Teams, recorded and transcribed verbatim using the record and transcribe option available on Microsoft Teams. Transcripts were verified by two research team members (R. N. and B. D. P.). Personal identifiers were removed from the transcript. The interviews were not time‐limited but rather led by the engagement of the participants.

### 2.3 Data analysis

The interviews were analysed using thematic analysis, as described by Braun and Clarke.^41^ Transcripts were read at least three times by two independent researchers (R. N. and B. D. P.) to identify initial patterns (familiarisation). Computer software NVivo^42^ was used to analyse the data. The codes identified were combined into possible themes, and the relationship between themes was considered (theme development). The final steps involved reviewing, defining and naming the themes, and forming a clear thematic map driven by the data. The preliminary data analysis was conducted by two research team members (R. N. and B. D. P.) and refined by a discussion with members of the research team (R. D. and C. G.). The clarity and trustworthiness of the data analysis and its reporting were further enhanced by the contribution of a parent and patient representative.

## 3 RESULTS

**Figure 1:**
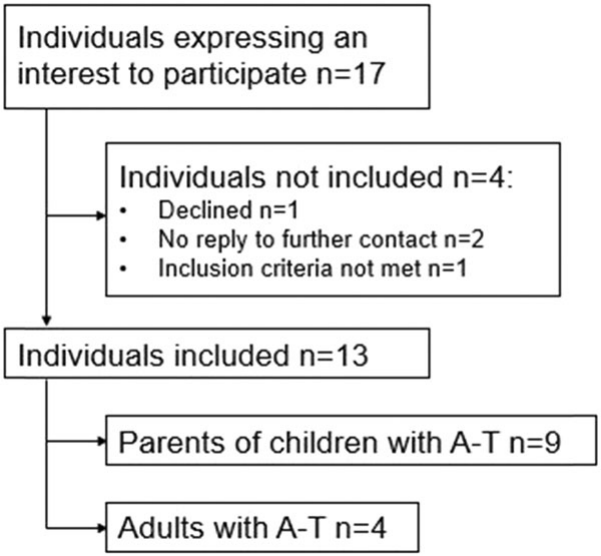
Flowchart explaining the recruitment process. A‐T, ataxia–telangiectasia.

No children with A‐T were recruited. Seventeen adults expressed interest in taking part in the study. One adult with A‐T declined to participate in the study after reading the participant information sheet due to the subject of the study and one parent was excluded due to not meeting the inclusion criteria. Two parents did not reply to further emails after expressing their interest in participating in the study. Nine parents of children affected by A‐T and four adults with A‐T were interviewed in 11 interviews. In two interviews, both parents were present at their request (Figure 1).

Interviews ranged between 20 and 50 min (mean = 34) for parents and 9–20 min (mean =  15) for adults with A‐T. The participants' characteristics are described in Table 1.

**Table 1:** Participant characteristics were included in the data analysis.

Characteristics	
**Parents**	
*Relation to child*	
Mother	6
Father	3
*Number of children with A‐T*	
One child	5
Two children	2
*Type of A‐T*	
Classic	7
Variant	1
Unsure	1
*A child with a previous diagnosis of cancer*	
Yes	2—acute lymphoblastic leukaemia —Non‐Hodgkins lymphoma
No	7
**Adults with A‐T**	
Mean age (years)	29 (SD = 6.56; age range = 23–36)
*Gender*	
Female	4
Male	0
*Type of A‐T*	
Classic	2
Variant	2
*Diagnosis of cancer*	
Yes	2
No	2

*Note*: Bold font indicates the two groups included: Parents of people with A‐T and Adults with A‐T.

Abbreviation: A‐T, ataxia–telangiectasia.

**Figure 2:**
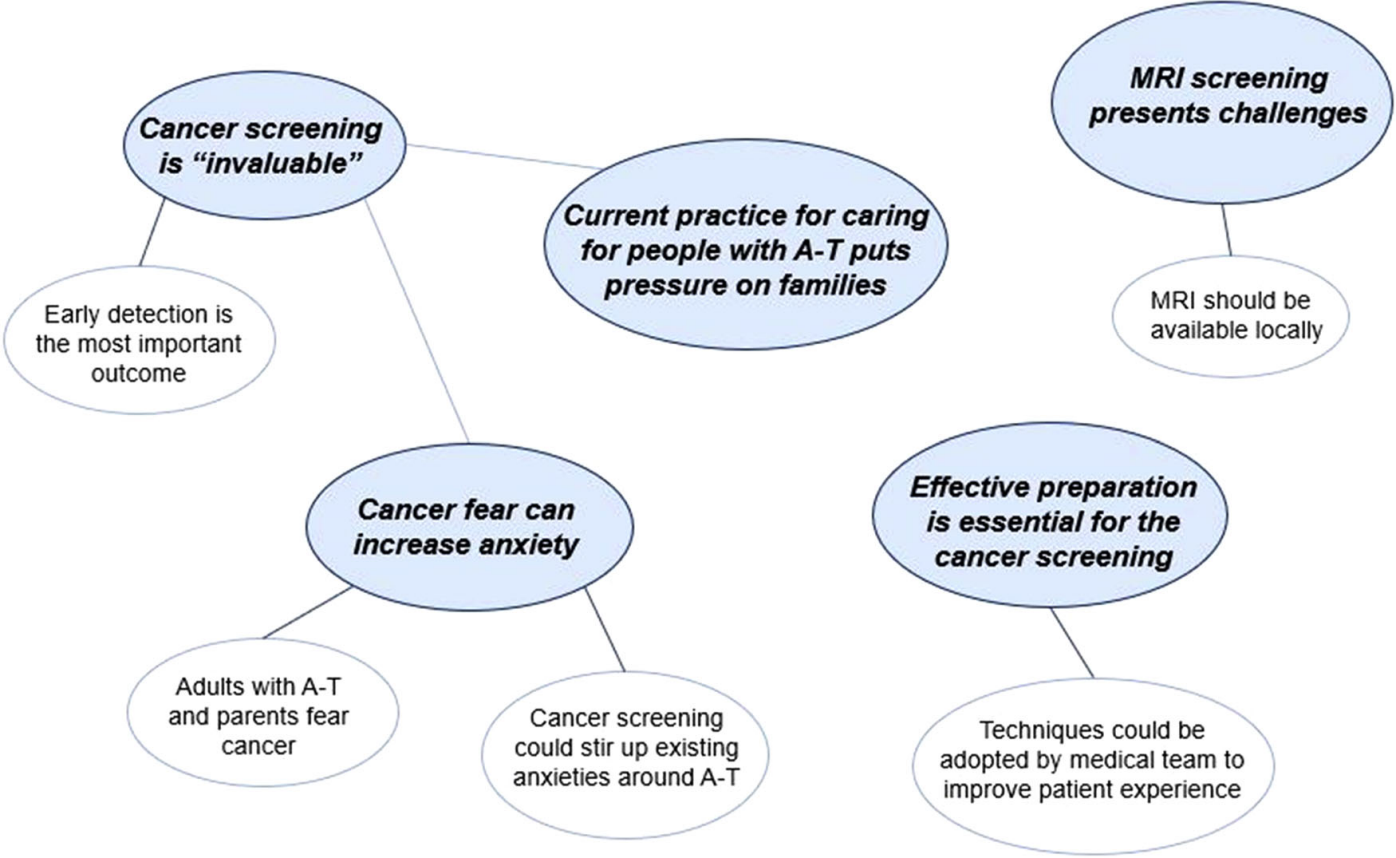
Thematic map showing the five main themes (Bold font) with the associated subthemes. A‐T, ataxia–telangiectasia; MRI, magnetic resonance imaging.

Five themes were identified from the data collected: (1) Cancer screening is ‘invaluable’, (2) Cancer fear can increase anxiety, (3) Current practice for caring for people with A‐T has limitations, (4) Effective preparation is essential for the cancer screening and (5) MRI screening presents challenges. Five subthemes were identified from four of the five main themes (Figure 2). The themes with illustrative quotes are shown in Table 2.

**Table 2:** Illustrative quotes of themes.

***Theme 1*** **Cancer screening is ‘invaluable’**	‘The more that's being done to protect them, the longer we have him with us’. (Parent_12) quote#1 ‘I think that would be everyone's reassured. You know, regular assurance that everything is still fine, everything's OK, and just allows you to kind of move on with life a little bit more’. (Parent_05) quote#2 ‘MRI as a tool, it seems like it's a safe one. Relatively safe because there's no radiation and it's not harmful, right? If it's not harmful for them and they're more, it seems like there are a lot more benefits than [negatives]’. (Parent_01) quote#3
*Subtheme* Early detection is the most important outcome	‘The earlier it's detected, the more reaction time you have, the better the prognosis (…) surely you know if you explained it to them that this would actually ‐ not necessarily cure, but possibly give a child fight, or any person, a fighting chance really’. (Parent_02) quote#4 ‘Part of me would say that's good [detecting any pathology], because they've caught whatever it is early. The other part of me was worried. About nothing in particular, just in your mind you want to run away with you, but at least you found it early’. (Adult AT_08) quote#5
***Theme 2*** **Cancer fear can increase anxiety**	‘From a parent's perspective, I am worried about cancer all the time, every day you know any small thing I'm like: “Oh my God!”, you know more anxiety than you would for a healthy child’. (Parent_09) quote#6
*Subtheme* Adults with A‐T and parents fear cancer	‘I think this [cancer] is one of the worst aspects of A‐T. (…) I have heard that a friend of mine's daughter died, who had A‐T with cancer. And she [her friend] said to me, her words were: cancer is the true face of A‐T, so it's the worst. It's the worst bit. because you can't do anything about it’. (Parent_09) quote#7
*Subtheme* Cancer screening could stir up existing anxieties around A‐T	‘You work quite hard to put the thoughts about the possibilities of A‐T at the back of your head, and then obviously, when you come to the screening, you would then be thinking about the possibilities [of a cancer diagnosis]. So, it's quite difficult emotionally to keep calm’. (Parent_09) quote#8 ‘Part of me would say that's good [to find any pathology, even not cancer‐related], because they've caught whatever it is early. The other part of me was worried. About nothing in particular, just your mind…but at least you found it early’. (Adult AT_08) quote#9 ‘Waiting [for the results] is always the worst thing. Doesn't matter who you are’. (Parent_07) quote#10
***Theme 3*** **Current practice for caring for people with A‐T puts pressure on families**	‘It's particularly the cancer field, there isn't anybody looking after these children. There's no one there to help the cancer side, if the child gets diagnosed with cancer. (…) There isn't anybody there. It's not like some of the other fields like neurology, and you know, you can go to this doctor, if you have immunology, you go to this doctor. There's no one there, so people are quite terrified of not just the cancer, but that there's nobody there to help’. (Parent_09) quote#11 ‘You know it feels like everything is in my hands, in our hands. And that's not a safe thought’. (Parent_01) quote#12
***Theme 4*** **Effective preparation is essential for the cancer screening**	‘The only possibility of it [cancer screening] being negative is if it is not dealt with sensitively in the way I explained, how you explain these things to the children. We don't want them to leave [the hospital], you know, paranoid that this thing is going to happen to them’. (Parent_09) quote#13 ‘I mean, are the other results of these MRI's seen immediately, or does it take a while to see? You know, once the MRI images are taken, is it on the spot that you can tell right away if something is happening, or would it go to like a doctor, who then reads out the result to you three months later?’ (Parent_01) quote#14
*Subtheme* Techniques could be adopted by the medical team to improve patient experience	‘Every time I go, they always make sure I'm comfortable. They gave me some headphones. They always talk to me and tell me what they're doing. Like they're going to start, going to change positions. I've had no problems’. (Adult AT_11) quote#15 ‘I think it's a frightening and alien‐looking machine. So, some sort of preparation would surely be beneficial to their mental, er, acceptance, yeah. Same with the parents. It's quite traumatic seeing your little girl disappear into the machine. We found that very hard’. (Parent_02) quote#16 ‘As a mum, I think, sometimes it's making sure the nurse that's doing the bloods is really used to doing them on children’. (Parent_07) quote#17
***Theme 5*** **MRI screening presents challenges**	‘Some people with A‐T might find it difficult to get onto the bed. And they might also find it difficult to be restricted. I think. Being restricted, it might be a bit claustrophobic, to some people’. (Adult AT_08) quote#18 ‘People with A‐T need to have the option to be anesthetised because it was very difficult for me to be awake and keeping still for all that time… This means that. I can't sit here and, uh, to move’. (Adult AT_08) quote#19 ‘We know obviously it's upsetting. I mean, because MRI means he's going to get knocked out basically for it to be done. I don't know how it is when they get older, but yeah, as children as far as I'm aware, they need to be under general anaesthetic, I think it was, and that is, that's upsetting’. (Parent_03) quote#20
*Subtheme* MRI should be available locally	‘Clearly, it will be more efficient if it could be local, rather than always having to go to, I don't know, Nottingham, Papworth, whatever it might be, or London hospital. It will be useful if that could be localised and I don't know if that's potentially possible, but that'd make it easier for parents to attend from a logistics point of view and also a cost, you know’. (Parent_07) quote#21

*Note*: The Bold font corresponds to the main themes.

Abbreviations: A‐T, ataxia–telangiectasia; MRI, magnetic resonance imaging.

### 3.1 Cancer screening is ‘invaluable’

Overall, the participants showed high expectations about cancer screening for people with A‐T. All participants expressed positive views about the prospect of a surveillance programme that screened for cancer (quote#1). In addition, some parents considered the regular monitoring that the cancer surveillance offers as proactive care, which is how they would like to see the health system working (quote#2). The families expressed positive views about the safety of MRI, in terms of avoiding the use of ionising radiation (quote#3).

#### 3.1.1 Early detection is the most important outcome

Early detection was the main anticipated advantage of cancer screening mentioned by most participants, on the assumption that it could lead to earlier treatment and improved outcomes. Participants emphasised that if cancer is detected too late, the person with A‐T could experience worse health outcomes (quote#4). Participants reported that cancer surveillance might lead to the detection of other pathologies that might not be related to cancer, which was seen as an advantage of cancer surveillance (quote#5).

### 3.2 Cancer fears can increase anxiety

This theme captured the strength of fear participants felt about the risk of cancer associated with A‐T (quote#6).

#### 3.2.1 Adults with A‐T and parents fear cancer

Participants reported that a diagnosis of A‐T is a stressful and life‐changing event on its own. In addition, with this diagnosis comes the awareness of the increased risk of cancer associated with A‐T and the lack of treatment options. Therefore, parents are constantly worrying about cancer (quote#7).

#### 3.2.2 Cancer screening could stir up existing anxieties around A‐T

When discussing the implications of having a cancer screening programme in place, the participants explained how they would experience some anxiety before the diagnostic test, not only because of the implications of the results but also because it is a reminder of the A‐T condition itself (quote#8 and quote#9). The participants discussed the fact that they could experience anxiety waiting for the results of the cancer screening. However, because they need to attend several medical appointments for other A‐T‐related issues, some participants mentioned that anxiety is often present in their lives and thus they have learned how to deal with these feelings (quote#10).

### 3.3 Current practice for caring for people with A‐T puts pressure on families

Participants discussed a range of medical specialists involved in the care of people with A‐T but reported a lack of oncological support (quote#11). The parents (particularly) reported constantly looking after their children's health, suggesting that they felt as if they were acting as doctors (quote#12).

### 3.4 Effective preparation is essential for the cancer screening

This theme captured the need for tailored communication with age‐appropriate education and information before cancer screening.

The participants believed that attending a medical centre/hospital for cancer screening would be acceptable for people with A‐T as they are used to having medical appointments. However, they reported the need to improve awareness about cancer screening. Parents need to be provided with information and education about the purpose of the screening before the MRI scan. Parents reflected that the support given to people with A‐T should be tailored to their age, experience, knowledge and background. Therefore, the information should be provided in layperson's terms (quote#13). When asked about what information should be provided, the participants highlighted the importance of knowing the technical aspects associated with cancer surveillance (such as the range of cancers that can be detected), the frequency of screening and the length of time to receive the results (quote#14).

#### 3.4.1 Techniques could be adopted by the medical team to improve patient experience

Participants identified critical aspects that can help people with A‐T to undergo an MRI scan: the use of a mock scan, listening to music, watching a movie and allowing their parents in the MRI room. They indicated that the medical staff should be friendly and calm. An adult with A‐T reflected upon her experience having an MRI scan, which was improved by having frequent communication with the radiographer during the scan (quote#15). The influence of past experience on how the person with A‐T would feel about having an MRI scan was discussed, with most having previously had a scan, which might make them feel more comfortable with the procedure. However, the medical team should be aware of this to ensure they provide the necessary support (quote#16). Regarding blood tests, participants discussed that people with A‐T have regular blood tests and thus they learn how to tolerate these tests. They believed that healthcare professionals should be experienced with the procedure and explain how the blood test will be performed (quote#17).

### 3.5 MRI screening presents challenges

This theme captured the challenges and some concerns regarding the practical and emotional aspects of the MRI scan.

The participants expressed some concerns related to the practical aspects of having an MRI scan, such as claustrophobia, laying still, the noise, the length of the scan and the need for anaesthesia for some people (quote#18). Particularly, a participant with A‐T felt that from personal experience, people with A‐T should have the option to be anesthetised as it was quite difficult for her to lay still in the MRI scan, as the results could be inconclusive due to excessive movement (quote#19). However, the parents expressed worries related to the implications of being anaesthetised, which for them is one of the most concerning aspects of the cancer surveillance programme (quote#20).

#### 3.5.1 MRI should be available locally

Participants expressed that having the MRI available locally would provide a better experience by minimising the challenges of travelling to a distant centre. Nevertheless, they reported that they would be willing to travel far if that ensured they could have the cancer screening (quote#21).

## 4 DISCUSSION

This study explored the views and feelings of both people with A‐T and parents of children with A‐T regarding the use of WB‐MRI for cancer surveillance in A‐T. Our results show that cancer screening in A‐T is perceived as valuable and desirable by people with A‐T and their families. The participants highlighted early cancer detection as the main perceived advantage of cancer screening protocols, which could allow earlier initiation of treatment. Participants mentioned the emotional comfort that comes with proactive, regular medical checks, and feeling they are doing something positive for their health or their child's health. This is important because they feel there is a lack of support in A‐T regarding oncology. This could be particularly relevant following the COVID‐19 pandemic, where medical consultations are increasingly being performed remotely as telephone or video consultations, reducing the opportunity for a medical professional to physically examine the person with A‐T. These results align with previous studies that evaluate the psychosocial impact of cancer surveillance in people affected with Li‐Fraumeni. They indicated that overall participation in the cancer surveillance programme does not bring additional psychosocial burdens to people with LFS.^36^, ^37^ In fact, the regular monitoring implied in a cancer screening provides a sense of control to people with LFS.^36^, ^37^

Participants felt that screening could temporarily increase their levels of anxiety before the screening test, during the scan and while waiting for the MRI results. This was balanced by the recognition that living with A‐T is already emotionally demanding and as a result, they had built up the resilience needed to cope with any additional short‐term anxiety. These results are in accordance with previous studies showing that the highest anxiety levels are at baseline but then are reduced a few weeks after the scan, especially after getting the results.^36^, ^37^ The concept of ‘scanxiety’ is recognised in the oncology field as the feeling related to the anticipation of having the scan and its results.^20^, ^43^

Some potential strategies to manage the anxiety and concerns associated with cancer surveillance protocols were identified. Participants highlighted the importance of having continuous communication and support from their medical team, which should be tailored to their knowledge and experience. An important note that needs to be considered is that the speech in people with A‐T is often affected, therefore the medical consultation should not be time restricted and perhaps should have a family member or a friend who can facilitate the communication. The participants mentioned the importance of ongoing education and information about the cancer risk in A‐T and the advantages of adhering to cancer screening guidelines. This is in line with previous studies that mentioned regular appointments as an opportunity for families affected by LFS to express their worries and to receive appropriate support.^36^, ^37^ The parents in our study expressed some concerns about communicating information to their children without causing worry. Previous studies suggested that regular appointments associated with cancer screening protocols allow ongoing education and communication with their medical team, which can help the parents to provide detailed and age‐appropriated information to their children about their condition.^38^, ^44^

Parents in this study reported that they face difficulties knowing when and how to discuss the cancer risk associated with A‐T with their children. It is therefore important that health professionals are able to make use of the best practice to build parents’ confidence. For example, the World Health Organisation guideline for HIV counselling has highlighted the psychological and health benefits of disclosure to children and outlines evidence‐based strategies to improve the disclosure process.^45^ MacMillan Cancer Support is another organisation which provides helpful support and reassurance regarding health education for children and young people with potentially life‐limiting conditions.^46^ A previous study that explored the parent‐child communication in genetic testing for LFS concluded that these discussions could be a source of distress and therefore the medical team should provide individualised support for the families, taking account of factors such as level of cognitive function and previous experience.^44^ This study suggested that going to surveillance appointments allows these discussions to occur naturally, which becomes more important during adolescence when individuals start to develop greater awareness and involvement in the management of their condition.

Participants suggested that detailed information about technical aspects of a cancer screening programme, such as the frequency of screening, the length of time to receive the results and the diagnosis performance of WB‐MRI scans, needs to be discussed before participation in a cancer screening programme. This information would help to reduce the anxiety levels experienced before participation and enable informed decision‐making. These results are highlighted in previous trials that study the psychosocial impact of using WB‐MRI for cancer surveillance in people with LFS.^36^

The participants mentioned challenges associated with WB‐MRI, such as the length of the scan, the noise of MRI scans and the need for anaesthesia for some people. They emphasised that preparation is the key to helping their children to undergo the MRI scan. The value of adequate preparation for children undergoing MRI scans is confirmed in the literature.^47^, ^48^ Viewing an internet‐based, animated video before an MRI scan was associated with a good level of knowledge about the MRI procedure and lower levels of anxiety before scanning.^49^ It is important to note that previous studies that assessed the use of WB‐MRI for cancer surveillance in people with LFS demonstrated that this approach is well tolerated.^27^, ^28^, ^29^

### 4.1 Study limitations

This study has limitations that should be considered. First, the adults with A‐T interviewed were all female, so our study does not have the perspectives of male adults with A‐T. Second, we did not have the chance to assess the feelings of children with A‐T between twelve and eighteen years old about cancer surveillance. Our difficulty recruiting people with A‐T in this age group may have been due to parental reluctance to bring their children into discussions on this sensitive and potentially upsetting topic. Thirdly, overall recruitment was lower than expected, possibly due in part to the COVID‐19 pandemic, as we had hoped to recruit through parent workshops and A‐T family events. Although online interviews offered convenience, and participants were provided with details of additional support in our patient information sheet, it may have been more acceptable and appropriate to recruit to face‐to‐face interviews in supportive settings. However, a sample size of 13 exceeds the suggested minimum of 12 interviews necessary to ensure data saturation (39).^40^ A larger and more diverse sample may have revealed other views, but we believe the risk of this is low given the small target population and the evident data saturation achieved in our analysis. Finally, we gathered the perspectives of parents and people with A‐T about the hypothetical use of WB‐MRI for cancer screening before participating in such a programme. Thus, their views may differ after undergoing surveillance. However, it is important to note that we aimed to understand their views before conducting a feasibility trial to have their perspectives implemented in the trial design.

## 5 CONCLUSION

In summary, our results suggest that WB‐MRI for cancer screening will be accepted and welcomed by people with A‐T and their families. The responsibility and pressure of managing a life‐limiting condition such as A‐T was acknowledged by participants in this study, and our results suggest that parents and people with A‐T perceive that the benefits of early cancer detection would outweigh the additional psychological burdens of screening. In addition, parents and adults with A‐T were able to suggest strategies to reduce the psychological impact of a cancer surveillance programme, including ongoing clinical support and continuous communication to provide psychosocial care.

## AUTHOR CONTRIBUTIONS

*Study conception and design*: Robert A. Dineen, Cris Glazebrook, Rafal Panek, Sophie Wilne, Mohnish Suri, William Whitehouse, Sumit Jagani, Madhumita Dandapani. *Recruitment*: Renata Neves, Blanca de Dios Perez, Tierney Tindall. *Data collection*: Renata Neves, Blanca de Dios Perez, Tierney Tindall. *Analysis and interpretation of results*: Renata Neves, Blanca de Dios Perez, Madhumita Dandapani, Robert A. Dineen, Cris Glazebrook. *Draft manuscript preparation*: Renata Neves. *Manuscript review and editing*: Renata Neves, Robert A. Dineen, Cris Glazebrook, Rafal Panek, Sophie Wilne, Mohnish Suri, William Whitehouse, Sumit Jagani, Madhumita Dandapani, Blanca de Dios Perez, Tierney Tindall, Natasha Schneider Fernandez.

## CONFLICT OF INTEREST STATEMENT

The authors declare no conflict of interest.

## Supporting information

Supplementary information.

Supplementary information.

Supplementary information.

## Data Availability

The data that support the findings of this study are available on request from the corresponding author. The data are not publicly available due to privacy or ethical restrictions.
