# Supplementary material for: Whole‐body MRI for cancer surveillance in ataxia–telangiectasia: A qualitative study of the perspectives of people affected by A‐T and their families
Source: Health Expect. 2023 Mar 16;26(3):1358–67. doi: 10.1111/hex.13756 (PMC10154855; doi:10.1111/hex.13756)
Supplement: Supplementary file 1 — Supplementary information. [file HEX-26-1358-s002.docx]

**Whole-Body MRI for cancer surveillance in Ataxia Telangiectasia: A qualitative study of the perspectives of people affected by A-T and their families**

**Supplementary file**

*Supplementary material S1*

Link of the video presentation of our research study.

<https://youtu.be/iEhiC8aTbUM>
