# Supplementary material for: Whole‐body MRI for cancer surveillance in ataxia–telangiectasia: A qualitative study of the perspectives of people affected by A‐T and their families
Source: Health Expect. 2023 Mar 16;26(3):1358–67. doi: 10.1111/hex.13756 (PMC10154855; doi:10.1111/hex.13756)
Supplement: Supplementary file 2 — Supplementary information. [file HEX-26-1358-s001.docx]

**Whole-Body MRI for cancer surveillance in Ataxia Telangiectasia: A qualitative study of the perspectives of people affected by A-T and their families**

**Supplementary file**

*Supplementary material S2*

**Interview Schedule, Group A** – Parents / carers of people with A-T (Focus Group or Interview)

**Understanding the views of people affected by ataxia-telangiectasia on the use of MRI screening to detect cancer**

- Welcome and introduction by researchers about themselves and their experience of A-T and any relevant personal professional information.
- Brief explanation of the purpose of the focus group / interview
- A reminder that participants can talk to researchers one-to-one after the focus group, if there are any points that they don’t want to make in from of other participants.

**Focus group / Interview Questions for Group A**

1. Opening question: Could you introduce yourself (to the group)? What is your connection to A-T?
2. What do you think about MRI scans as a screening tool? How do you think children might feel?
3. How do think parents of children with A-T feel about their children’s risk of cancer?

Prompts: How much of a concern to you is it? Is it something that you think about often?

1. How would you feel if more reliable routine cancer screening involving combining blood tests with regular routine MRI scanning was available?
2. What would you want to know about screening before you agreed that you or your child could take part in the screening programme?
3. What would be the impact on your child with A-T of taking part in screening? Any emotional impacts?
   1. What about you? How would you feel?
   2. What about other members of the family? How would they feel?
4. If a formal cancer screening programme for children and young people with A-T was introduced, would you use it?

Prompts: What would make you use the programme? What would put you off using the programme?

More specific questions that could be asked if not covered in the discussion:

1. Do you think that taking part in a cancer screening programme would have any negative effects on your family member with A-T or other family members?

Prompts: What negative impacts can you foresee? Are you concerned that taking part could cause anxiety? If so, would that anxiety be in the run up to the test, or while waiting for the result? Are you concerned about the test causing anxiety for the person with A-T, other family members, or both?

1. Do you think that taking part in a cancer screening programme would have any positive effects on your family member with A-T or other family members?
2. Would you have concerns about the possibility of a positive diagnosis of cancer (i.e. the need for further tests and treatments)?

Prompts: What are the particular concerns? Are these concerns sufficient to make you consider whether taking part in a cancer screening programme are worth it?

1. How would you feel if there was a chance that the MRI scan might find things that needed further investigation but turned out *not* to be cancer?

Prompts: Would that potentially put you off participating?

1. How often would be too often for you / your family, in terms of attending your local Children’s Hospital for cancer screening scans?

Prompts: For example, would once a year be acceptable? How about if every 6 months was the recommendation?
