# Supplementary material for: Whole‐body MRI for cancer surveillance in ataxia–telangiectasia: A qualitative study of the perspectives of people affected by A‐T and their families
Source: Health Expect. 2023 Mar 16;26(3):1358–67. doi: 10.1111/hex.13756 (PMC10154855; doi:10.1111/hex.13756)
Supplement: Supplementary file 3 — Supplementary information. [file HEX-26-1358-s003.docx]

**Whole-Body MRI for cancer surveillance in Ataxia Telangiectasia: A qualitative study of the perspectives of people affected by A-T and their families**

**Supplementary file**

*Supplementary material S3*

**Interview Schedule, Group B** – Children and young people with A-T (Interview, with parent or carer as appropriate)

**Understanding the views of people affected by ataxia-telangiectasia on the use of MRI screening to detect cancer**

- Welcome and introduction by researchers and participants
- Brief explanation of the purpose of the interview

**Interview Questions for group B**

1. Opening question: What is your favourite movie? (this can be linked to watching a film while having the MRI scan)
2. Have you already had an MRI scan?

2a. If yes, How did you find the experience?

2b. If not, how would you feel about having an MRI scan?

Prompts: how did / would you find having to lie very still? Did / would the loud noises concern you?

1. How would you feel about having an MRI scan every year so that we can monitor your heath?

Prompts: Is there anything that would worry you about having a scan? Is there anything that would make you more happy to have a scan? How would you feel about the extra visits to the hospital? What would make these visits more pleasant?

1. How would you feel about having a blood test every year so that we can monitor your health?

Prompts: Is there anything that would worry you about having a blood test? Is there anything that would make you more happy to have a blood test?

1. The scan or blood test might show things (use the word ‘cancer’ if the parent has agreed to this) that need to have extra treatment. How would you feel about having a scan or blood test that could find something that needs extra treatment?
